# The senescent cell epigenome

**DOI:** 10.18632/aging.101617

**Published:** 2018-11-03

**Authors:** Na Yang, Payel Sen

**Affiliations:** 1 National Institute on Aging, NIH, Laboratory of Genetics and Genomics, Functional Epigenomics Unit, Baltimore, MD 21224, USA; 2 Epigenetics Institute and Department of Cell and Developmental Biology, University of Pennsylvania, Smilow Center for Translational Research, Philadelphia, PA 19104, USA

**Keywords:** chromatin, epigenetics, senescence, aging, histone

## Abstract

A critical hallmark of aging is cellular senescence, a state of growth arrest and inflammatory cytokine release in cells, caused by a variety of stresses. Recent work has convincingly linked the accumulation of senescent cells in aged tissues to a decline in health and a limit of lifespan, primarily through "inflammaging". Importantly, interventions that clear senescent cells have achieved marked improvements in healthspan and lifespan in mice. A growing list of studies show that environmental stimuli can affect aging and longevity through conserved pathways which, in turn, modulate chromatin states. This review consolidates key findings of chromatin state changes in senescence including histone modifications, histone variants, DNA methylation and changes in three-dimensional genome organization. This information will facilitate the identification of mechanisms and discovery of potential epigenetic targets for therapeutic interventions in aging and age-related disease.

## Cellular Senescence and Senotherapies

### Causes of senescence

Aging is accompanied by the accumulation of senescent cells in tissues. Cellular senescence was first discovered in cultured fibroblast cells, where prolonged passaging and replicative exhaustion led to growth arrest due to critically short telomeres [1,2]. Senescence was eventually also observed in cells subjected to a variety of stressors including oncogene induction, oxidative stress, acute DNA damage or mitochondrial dysfunction [3,4]. Senescent cells are characterized by (a) a cell-autonomous proliferative arrest [1], (b) expression of lysosomal beta-galactosidase [5], (c) resistance to apoptosis [6,7] and mitogenic signals, (d) release of inflammatory cytokines and chemokines (also known as senescence-associated secretory phenotype or SASP) [8], (e) persistent DNA damage [9] and (f) formation of senescence-associated heterochromatic foci (SAHF) [10] and changes in DNA methylation [11]. At the molecular level, growth arrest is initiated by DNA damage that signals the upregulation of CDKN1A/p21CIP1/WAF1 by p53 stabilization and binding to promoter of the p21 gene. An independent upregulation of CDKN2A/p16INK4A, an inhibitor of cyclin dependent kinases 4 and 6, cooperates with p21 to reduce the phosphorylation of retinoblastoma (Rb) protein and arrest cells at the G1 phase [4,12]. These upstream signals also maintain the active metabolic state of senescent cells while promoting marked changes in the gene expression program such as downregulation of cell cycle genes and upregulation of SASP genes [13,14].

A number of factors including DNA damage and dysfunctional mitochondria are causal to the senescent phenotype. For example, telomere shortening is the primary cause of replicative senescence (RS) resulting from exposure of telomeric ends due to removal of the Shelterin complex, and their recognition as double strand breaks [15]. Activation of oncogenes such as RAS or BRAF results in oncogene-induced senescence (OIS) by engaging three machineries; repression of pro-proliferative E2F target genes, replication stress-activated DNA damage response and SASP [16]. Mitochondria can modulate the senescence phenotype by two related mechanisms: hyperactivation of the TCA cycle and upregulation of SASP. Changes in the levels of TCA cycle metabolite malate in senescence is influenced by p53-mediated repression of malic enzyme 2 [17]. Senescence can be directly influenced by overexpressing or inhibiting specific enzymes in the TCA cycle [18]. In OIS, increased pyruvate oxidation due to elevated pyruvate dehydrogenase levels produces reactive oxygen species (ROS) that activates the DNA damage response. The DNA damage response triggers mitochondrial biogenesis through the ATM, Akt, mTORC1, PGC1ß pathway thus further increasing ROS production and genome damage. Selective ablation of mitochondria in senescent cells can reduce the pro-oxidative (ROS) and pro-inflammatory (SASP) arms of senescence [19].

Despite the causal triggers of senescence, their direct link to epigenomic changes is less clear. Studies from lower organisms provide key mechanistic insights that could influence the senescent cell epigenome. For example, mid-life flies show enhanced acetyl-CoA (an acetyl group donor to TCA cycle) levels, increased histone acetylation and longevity-altering transcriptome changes [20]. Along these lines, elevated pyruvate dehydrogenase levels in OIS cells also increase acetyl-CoA production and can potentially influence histone acetylation [21]. These findings provoke a theory that chronic DNA damage and altered metabolism in midlife can trigger the onset of senescence and ultimately tissue aging via chromatin [22]. However, the exact mechanisms remain to be tested.

Taken together, the anti-proliferative nature of senescent cells serves a potent tumor suppressive mechanism. However, the chronic DNA damage, ROS and SASP promotes both local and systemic dysfunction at the tissue and organ level [23].

### Consequences of senescence

Senescent cells accumulate in aged tissues due to exhaustion of proliferation-competent cells and adult stem cells [24]. In age-related diseases, senescent cells are often found at sites of pathology [25–27] in mice and humans. This accumulation in turn, disrupts tissue homeostasis, reduces regenerative capacity and remodels the tissue micro-environment mimicking a chronic low-grade inflammation status with positive beta-galactosidase staining in multiple tissues, ultimately resulting in an irreversible structural and functional decline [13].

It is important to note that the total percentage of senescent cells in aged tissues is usually <20%. Quantitative estimation of beta galactosidase positive cells in aged murine tissues ranged from ~14% in subcutaneous stromal cells, 6.9% in lung epithelial cells, 3.3-3.5% in small intestinal and spleen cells respectively and 1.48% in lymph node cells [28]. In the skin of old baboons, senescent dermal fibroblasts were quantified to be ~15% [29]. In aged human skin, beta galactosidase positive keratinocytes and fibroblasts are notably higher in aged donors [5]. Although, the overall percentage of senescent cells in aged tissues seem low, it is important to remember that (a) their number correlates positively with age [5,28–30], (b) they release inflammatory cytokines capable of amplifying damage in surrounding tissue via a paracrine action [31] and (c) if senescence occurs in a particularly important fraction of cells, they can have deleterious consequences on tissue health and function [32].

### Targeting senescent cells to prevent age-related decline: senolytics and other senotherapeutic agents

There are multiple methods to improve senescence-related loss of function *in vivo* (see Future directions and translational perspectives). Of these methods, targeted elimination of senescent cells has shown remarkable promise [33]. In seminal studies, clearance of senescent cells extended the healthspan and lifespan of progeroid mice [27] and naturally aged mice [26]. Targeting senescent cells for elimination also improved tau-related pathology and cognitive loss in a mouse model of Alzheimer’s disease [34]. Administration of senolytic (agents that lyse senescent cells, primarily targeting an apoptotic mechanism specific to senescence [33]) cocktail dasatinib and quercetin improved physical function and lifespan in old mice [35]. Importantly, senescent cells causally affected age-related physical dysfunction as senescent cell transplants in young and old mice significantly decreased grip strength, walking speed, hanging endurance, etc. Local administration of senolytics has also shown marked functional improvement at atherosclerotic plaques and post-traumatic osteoarthritis [36]. Recently, a flavonoid polyphenol compound screen identified fisetin as another potent senolytic agent that is effective both *in vitro* and *in vivo* [37].

A number of pharmacological interventions targeting senescent cells are now in phase II/III clinical trials and include metformin, mitochondria-derived peptides and small molecule senolytics. Others such as rapamycin or JAK1/2 inhibitors show potent anti-SASP effects [38,39]. Rapamycin derivatives such as everolimus can also boost immune function in the elderly [40,41] and is in queue for clinical trials treating respiratory tract infections, heart failure and potentially boosting autophagy to prevent neurodegenerative diseases.

### Challenges and alternative approaches to developing new senotherapeutics

Despite monumental advancements in developing or repurposing drugs to target and kill senescent cells, the scientific community faces major challenges in designing therapies that are highly specific to the rare senescent cell population. Alternative approaches to senolytics will be to delay the onset of senescence altogether or restore senescent cells to their youthful state [33]. Senescent cells share similarities with terminally differentiated cells and one strategy to revert the bad effects of senescence is to induce dedifferentiation by overexpressing Yamanaka factors [42]. This method has achieved remarkable success both *in vitro* [42] and *in vivo* [43]. However, as an important note, these studies aim to only partially reprogram cells without re-entry into cell cycle. Since senescence is a potent tumor suppressor, mechanisms that provoke cell cycle re-entry can have deleterious pro-cancer outcomes [44]. An alternative safer strategy is to develop therapies that target epigenetic enzymes acting on the chromatin in senescent cells [45]. Although challenging, this strategy may be able to switch gene expression programs in senescent cells restoring youthful morphology, shutting down SASP and achieving metabolic balance.

The following sections discuss the accumulating evidence of chromatin changes in senescent cells both *in vitro* and *in vivo*. The goal of this review is to encourage the readers to identify emerging trends and devise novel epigenetic senotherapies to ameliorate age-related functional decline and disease. The final section of this review discusses senolytic alternatives and novel epigenetic approaches to prevent senescence-related damage in aged tissues.

## Chromatin Changes in Senescence

### Breaking the histone code in senescent cells

The histone code refers to the combinatorial patterns of posttranslational histone modifications primarily on the tails (but also on the globular domains) of basic histone proteins that form an integral part of chromatin [46]. There are four canonical histone proteins that constitute the histone octamer with two copies each of H2A, H2B, H3 and H4 forming a spool around which DNA is wound [47]. Additional diversity is provided by variant histones [48] (see below). A catalog of histone modifications on canonical and variant histones have now been identified using molecular biology, genomics and proteomic approaches including acetylation (ac), methylation (me), phosphorylation (p), ubiquitination (ub) among others [49]. Specific histone modifications are correlated with open/closed or active/repressed chromatin states [50]. For example, histone 3 lysine 4 trimethylation (H3K4me3) and H3K27me3 are common epigenetic modifications with opposing controls on transcription and which have been directly linked to longevity regulation in many systems [14,51]. H3K4me3 is an activating modification found at gene promoters and drive active transcription by RNA polymerase II. In contrast, H3K27me3 is a repressive modification that marks facultative heterochromatin. Senescence and aging in diverse organisms entails an imbalance of these activating and repressive histone marks as evidenced by global assessments such as western blotting, immunofluorescence as well as genomics-based profiling with consequences at the transcription level. However, there is no consensus on a working model that fully explains the effects of these activating and repressive changes on lifespan in all organisms [52].

### *Heterochromatic alterations: formation of SAHFs*

The quantitative assessment of the extent of imbalance of histone marks in senescent cells is confounded by declining levels of histone proteins triggered by DNA damage [53,54]. Despite this decline in total histone levels, distinct chromatin changes are visible by microscopy. OIS in human diploid fibroblasts is accompanied by a breakdown of the nuclear lamina (see below), loss of heterochromatin domains and large scale spatial rearrangements of chromatin, forming nuclear structures known as SAHFs [10]. SAHFs are formed by condensation of individual chromosomes into a single SAHF focus as identified by chromosome painting [55]. Confocal microscopy further elucidated a multilayer concentric structure of SAHFs with the repressive mark H3K9me3 enriched in the core and H3K9me2 covering the whole area of SAHF. In marked contrast to H3K9me3, H3K27me3 exhibited a “ring” structure, surrounding the H3K9me3 “core” separating it from H3K36me3-enriched transcriptionally active regions. In the genomic space, SAHFs coincide with late replicating regions. Furthermore, SAHFs are enriched in heterochromatic proteins HP1, chromatin architectural proteins of the HMGA family and histone variant macroH2A while excluding euchromatic marks such as H3K9ac, H3K4me3 and linker histone H1 [56]. SAHFs have been widely used as a marker of senescence with their mechanism of formation carefully mapped out. Prior to formation of SAHFs, HP1, HIRA and ASF1a (a chaperone complex for H3.3, see section on histone variants) transiently enter promyelocytic leukaemia (PML) bodies in a rate-limiting step. Subsequently, macroH2A is deposited onto chromatin and helps to stabilize SAHFs. These concerted events then promote cell cycle exit and durable growth arrest in senescence [57].

In addition to the microscopic studies that identified SAHFs, genomics analysis of a panel of histone modifications in growing and OIS cells, showed altered occupancy of active (H3K4me3 and H3K36me3) and repressive (H3K9me3, H3K9me2, and H3K27me3) histone marks with all except H3K9me3 being correlated with alterations in transcription. In addition, cluster analysis of ChIP signal of H3K9me3, H3K27me3 and H3K9me2 recapitatulated SAHF structure. However, although the marks were redistributed in some genic regions, the global pattern was highly static, suggesting that 3D repositioning rather than spreading of pre-existing H3K9me3 and H3K27me3 is involved in SAHF formation [56].

### *Genome-wide profiling of activating and repressive histone modifications: canyons, mesas and new enhancers*

A complementary study analyzing H3K4me3 and H3K27me3 distributions by ChIP-seq in RS, OIS and cells from Hutchinson Gilford Progeria Syndrome (HGPS) patients showed large-scale changes such as domains of H3K4me3 and H3K27me3-enriched “mesas” and H3K27me3-depleted “canyons”. Cells from HGPS patients therefore represent a population of “aged” but not necessarily true senescent cells unless passaged a few times *ex vivo*. Hereon, we refer to cells isolated from aged tissues as aged cells.

Mesas form at lamin B1-associated domains (LADs) while canyons mostly form between LADs and are enriched in genes and enhancers. Forced reduction of lamin B1 results in mesas and canyons. Localized H3K27me3 loss in canyons strongly correlates with upregulation of key senescence and anti-proliferation genes, including the canonical SASP genes [58]. In support, a decrease in the expression of the H3K27 methylase EZH2, a polycomb group protein, during RS and OIS directs a decrease of repression-associated H3K27me3 and rapid senescence in primary human cells, in part through upregulation of p16 [59]. On the contrary, inhibition of the H3K4 methylase MLL1, decreased the levels of H3K4me3 modestly and Υ-H2AX dramatically over SASP gene bodies, with a concomitant inactivation of their gene expression. Υ-H2AX is a variant histone deposited on DNA damage in SASP genes suggesting that MLL1 is a key regulator of the secretory phenotype through a DNA damage response pathway [45].

Other histone modifications implicated in senescence include H4K20me3 (repressive), H4K16ac and H3K27ac (activating). H4K20me3 is enriched in senescent cells and aged cells and especially at SAHFs with H3K9me3 which recruits the cognate H4K20 methylase SUV420H2, as well as at specific non-genic and genic repeats [60–63]. In contrast, RS cells show strong enrichment of H4K16ac at promoter elements of all expressed genes and its retention is dependent of histone chaperone HIRA [64]. Like H4K20me3, global levels of H4K16ac do not strongly correlate with gene expression changes in senescent cells. A similar pattern of H4K16ac enrichment was also observed with aged neurons in human brain samples [65]. A systematic profiling of H3K27ac in proliferating, quiescent and OIS cells revealed the dynamic remodeling of the regulatory enhancer landscape in senescence. Loss of narrow typical enhancers adjacent to the promoters of proliferation genes correlated with the shut-down of proliferation in senescent cells. In contrast, the formation of new super enhancers in senescence occurs close to genes related to the SASP program. A subset of these super enhancers are bound by BRD4, a bromodomain containing protein. BRD4 inhibition by BET inhibitors did not bypass senescence but specifically modulated SASP levels and reduced immune-surveillance both *in vitro* and *in vivo* [66].

**Figure 1:**
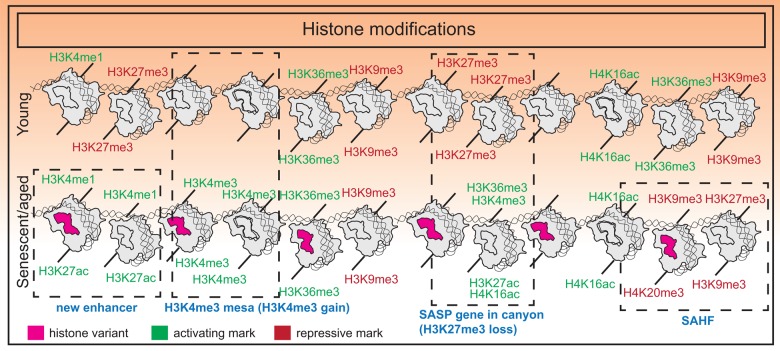
**Histone modification changes in senescence.** Senescence is associated with an imbalance of histone modifications with a tendency towards accumulating euchromatin marks. Additional features include formation of new super-enhancers near SASP genes in OIS, H3K27me3 “canyons” where SASP genes reside, H3K4me3 “mesas”, and formation of SAHFs.

Taken together, the chromatin landscape in senescent cells presents a unique environment that promotes formation of features such as SAHFs which reinforce a tumor suppressive phenotype, as well as large regulatory elements that activate SASP programs. Interestingly, the breadth of H3K4me3 domains and enhancer score are important predictors of aging in murine tissues as identified using machine-learning models [67]. Overall, the balance in activating and repressive marks is tipped towards an “opening” of chromatin structure that likely promotes genome instability while maintaining the senescent transcriptome. A summary of histone modification changes is shown in Figure 1.

### *Variant histones and altered nucleosomal composition in senescence*

Histone variants are non-allelic counterparts of the core canonical histone proteins varying in one or few amino acids located primarily in the C terminus. Generally, genes encoding the canonical forms of H3 (H3.1 and H3.2) contain one exon, lack introns, have a stem loop terminator and are not polyadenylated. Hence they undergo rapid turnover in contrast to variant histones. Additionally, canonical histones are synthesized only in the S phase whereas variant histones are made constitutively throughout the cell cycle in a replication-independent manner [68,69]. Thus, in non-cycling senescent cells, canonical histone production declines (in part due to reduced synthesis and in part due to high turnover) and variant histones tend to accumulate [53,64]. However, expression of a small subset of two-exon histone genes encoding of H2A, H2B and H4 are upregulated in senescence, allowing the formation of nucleosomes with altered composition [64].

One variant of histone H3 is H3.3, which differs from H3.1 and H3.2 (the canonical counterparts) at five and four amino acid positions respectively [69]. RS cells show strong expression of H3.3 and incorporation at promoter elements of active genes by ChIP-seq. H3.3 peaks overlap with HIRA, a chaperone responsible for H3.3 and H4 deposition on chromatin. HIRA is also required for the steady state maintenance of the histone modification H4K16ac, which largely overlaps with H3.3 peaks in senescent cells. These events then maintain senescence-related gene expression and enforce tumor suppression [64]. In RS and OIS cells, the deposited H3.3 can undergo N-terminal tail cleavage at two sites by the lysosomal protease Cathepsin L1. This processing removes histone N-terminal posttranslational modifications such as H3K14ac, H3K18ac, H3K4me3 etc. that critically control the expression of cell cycle regulators. HIRA-mediated loading of cleaved H3.3 is sufficient to promote cellular senescence [70].

MacroH2A isoforms (macroH2A1.1, macroH2A1.2 and macroH2A.2) are variants of H2A that have a 30KDa “macro” domain at their C-terminus. MacroH2A1 is found predominantly in heterochromatic regions such as the inactive X chromosome [71]. In senescent cells, macroH2A1 is localized to SAHFs. Although not critical for SAHF formation, macroH2A1 serves a maintenance role to stabilize SAHFs [57]. In RS cells, the poly-ADP-ribose (PAR) binding isoform macroH2A1.1 (but not PAR-binding deficient isoform macroH2A1.2) becomes enriched as cells exit the cell cycle [72]. Another study in OIS cells, showed an intricate model of macroH2A1 regulation of SASP genes. In proliferating cells, macroH2A1 binds to transcriptionally inactive SASP genes where it likely poises SASP genes for activation. Upon senescence induction by oncogenes and the initial burst of SASP activity, macroH2A1 is required for autocrine and paracrine effects of SASP. Sustained SASP however, allows for activation of the ER stress signaling and DNA damage response, elevating ATM activity, which in a negative feedback loop removes macroH2A.1 and repression of SASP genes [73,74].

The histone variant H2A.J differs from canonical H2A protein by the presence of an SQK motif near the C-terminus and an A11V substitution in the N terminal tail. Mass spectrometric studies showed that H2A.J accumulates in RS and DNA-damage induced senescence increasing in amount by almost ten-fold. Additionally, elevated H2A.J was found in senescent keratinocytes upon carcinogen treatment as well as in hair follicle stem cells and interfollicular epidermal cells of old mice, irradiated mice and aged human epidermal cells. Depletion of H2A.J by RNAi was found to reduce the expression of genes encoding proteins bound to the cell surface and those involved in the SASP response. However, this response could not be explained by differential incorporation of H2A.J in SASP gene promoters as measured by ChIP-seq, begging further analysis [75].

Overall, the abundance of variant histones (over canonical histones) during senescence promotes a permissive chromatin for establishment and maintenance of the senescent state.

### Profound alterations in the aging DNA methylome

DNA methylation is a second type of epigenetic control that involves either cytosine-5 methylation (5mC) within CpG dinucleotides or adenine-6 methylation (6mA). 5mC is a prominent and extensively studied modification in mammalian systems whereas other commonly studied aging models such as worms and flies either lack or have limited DNA methylation. DNA methylation is established during development through the action of de novo methylases DNMT3A and 3B, while DNMT1 plays a maintenance role [76]. Conversely, the ten-eleven-translocation (TET) proteins (TET1-3) mediate the removal of the methyl group in a three step iterative oxidation process, an intermediate of which is 5-hydroxymethyl cytosine (5hmC) [77]. 5mC and 5hmC together make up the bulk of methylated DNA with other oxidative products such as 5-formyl cytosine (5fC) and 5-carboxyl cytosine (5caC) being an order of magnitude less abundant [78].

5mC is recognized by a host of methyl CpG-binding domain (MBD) proteins including MBD1-4 and MeCP2 and together with co-repressors, prevent transcription from genes [79]. 5hmC on the other hand has been shown to be enriched in promoters, gene bodies and enhancers surrounding transcription factor binding sites and correlates with active transcription [80]. The genome-wide profiles of 6mA or 5hmC have not been characterized in senescent cells.

### *Alterations in the global distribution of 5mC*

Senescence and aging markedly alter the DNA methylation (5mC) landscape with global DNA hypomethylation co-occuring with focal hypermethylation [11,14]. Hypomethylation occurs primarily in repetitive regions (LINEs and SINEs) or late-replicating pericentromeric satellites and lamin-associated domains in the genome that normally correlate with constitutive heterochromatin. In senescent cells, one consequence of hypomethylated DNA at repeat regions of the genome is distension (senescence-associated distension of satellites or SADS) and derepression [81]. For example, in both RS and OIS cells, SADS can be visualized via 3D DNA fluorescent in situ hybridization (FISH) experiments on pericentric satellite II and centromeric alpha satellites [13,82,83]. FAIRE (formaldehyde assisted isolation of regulatory elements) data from RS cells in addition showed that chromatin from major retrotransposon classes, Alu, SVA and L1 become more open, ultimately resulting in more transcription and transposition during deep senescence [84]. Increased retroviral repeat element transcription is also evident in aged mouse heart, liver, cerebellum and olfactory bulb [67]. Hypermethylation occurs at promoter CpGs, and mostly correlates with gene repression. In RS, whole genome bisulfite sequencing revealed hypermethylation at promoters of gene related to cell cycle and tumor suppressors suggesting that the senescent DNA methylome may sensitize aged cells to malignancy. Genome-wide, differentially methylated regions (DMRs) in senescence, senescence-bypass and cancer showed partial but significant overlap. Importantly, methylation changes retained in bypass cells (compared to senescent cells) were enriched for methylation changes in cancer [11]. In RS, expression of DNMT1 and DNMT3B is downregulated [11], as well as TET1 and TET3 [85] and therefore cannot predict the direction of DNA methylation change. However, hypomethylation was shown to occur even in proliferation competent near-senescent cells suggesting a failure of DNMT1 to maintain methylation. This observation, together with the lack of DNMT1 nuclear puncta in senescent cells and premature senescence induced by DNMT1 knockdown strongly supports a dominant role of the maintenance methylase in driving the senescent phenotype [11]. DNA methylation changes are also a feature of tissue aging. Comparative analysis of the DNA methylome in young and old mice livers revealed that hypomethylated DMRs were enriched at intragenic enhancers in highly expressed liver-specific genes and hypermethylated DMRs were enriched at bivalent CpG islands [86,87]. Taken together, senescence and aging involves a bidirectional epigenetic drift in the DNA methylome that contributes to cellular dysfunction and likely cancer progression.

### *Development of a DNA methylation-based epigenetic clock*

Regression modeling of the methylation status of a set of CpGs has inspired the development of an “epigenetic clock”, serving as a robust biomarker of biological aging [88–90]. In the original study from UCLA, Horvath developed a multi-tissue predictor of age using 8000 samples from 82 Illumina DNA methylation array datasets, encompassing 51 healthy tissues and cell types [89,90]. He discovered that DNA methylation at 353 CpGs and no other epigenetic modifications provided an accurate age estimate. In a parallel study, Hannum et al performed genome-wide methylomic profiling of whole blood taken from a large cohort of individuals spanning a wide age range and variety of races. The study identified 71 CpGs as highly predictive of age [91]. Although the “clock” varies markedly across species and tissue types, several studies have refined CpG signatures to include a smaller subset that predict age independently of sex, tissue type, disease state and array platform [88]. Additionally, the "clock" has now been accurately defined in specific cell types and tissues for researchers working with those models. For example, mice liver “clocks” have shown that longevity altering interventions such as rapamycin and dietary restriction affects the biological age [86]. The “skin and blood clock” accurately predicts age for a variety of skin and blood cells and shows rapid age acceleration when cultured *ex vivo* [92]. Interestingly, the “clock” has now been commercialized as a direct-to-customer product by Zymo Research (sold as My Dnage) for predicting biological age in humans.

It is important to note that although the “epigenetic clock” correlates with cell passage, it is not a marker of cellular senescence. In one study that investigated RS, OIS and irradiation-induced senescence (IR), it was interestingly noted that RS and OIS cells were aged (as measured by the epigenetic clock) but not IR cells. This observation implies that DNA damage does not cause cellular aging and that cellular aging and senescence are uncoupled [93].

**Figure 2:**
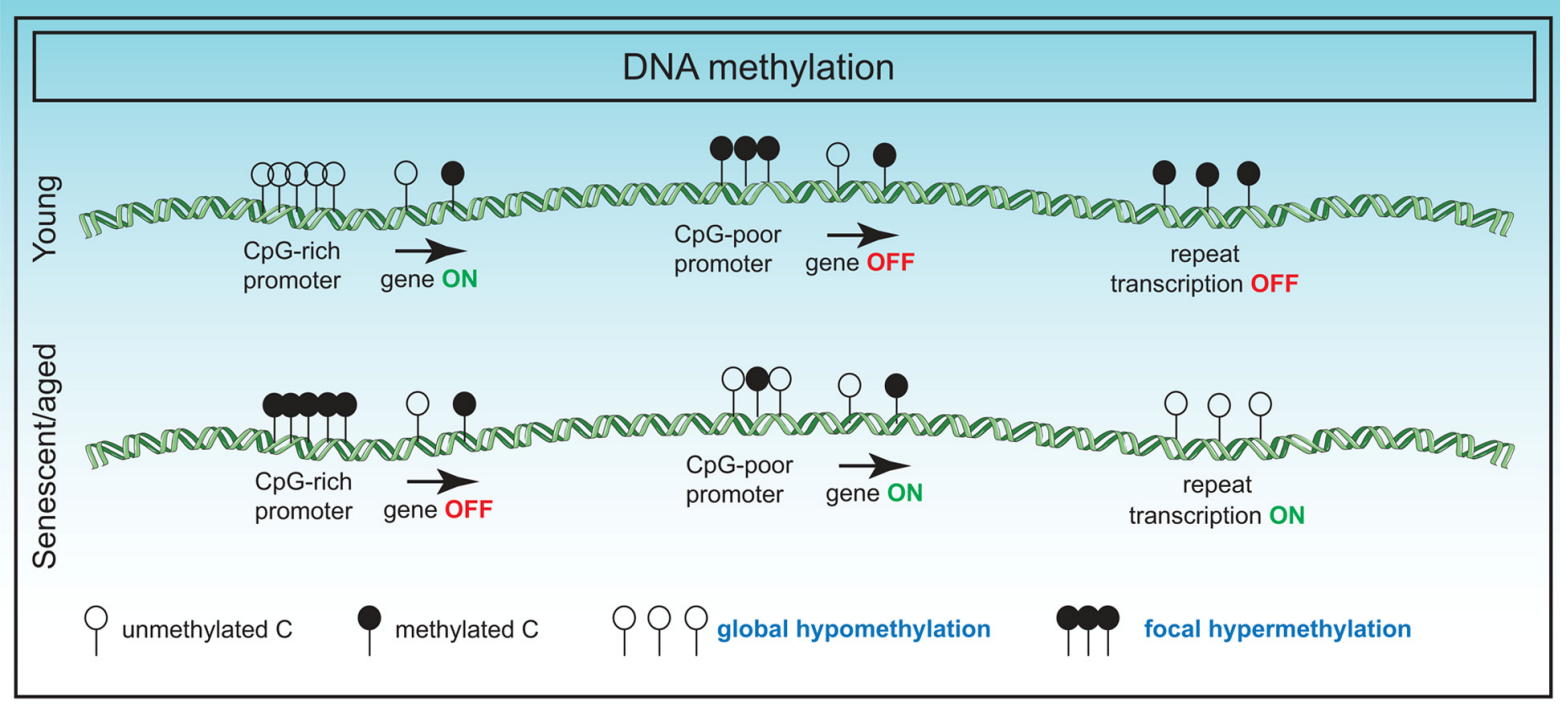
**Changes in the DNA methylome in senescence.** DNA methylation changes in senescence primarily involve global hypomethylation particularly at repeat regions and focal hypermethylation at CpG rich promoter sequences. These changes have adverse effects on gene expression.

Despite the accuracy of the “clock”, the mechanisms accelerating or slowing down the "clock" are not clear and in conjunction with other epigenetic predictors, would be a fascinating area of study. Additionally, the underlying biological meaning of the "clock" is also unknown, and genes associated with the clock CpGs show no differences in age-related gene expression with two exceptions. A genome-wide association study (GWAS) revealed specific variants inside a putative RNA helicase *DHX57* and near mTOR complex 2 gene *MLST8*, had cis-effects on gene expression in the cerebellum [94]. However, the apparent general lack of correlation with gene expression suggests that the "clock" is perhaps a highly sensitive readout of upstream signals such as hormonal changes or immune signaling resulting in 3D spatial or phase changes inside the nucleus. A summary of DNA methylome changes in senescence is summarized in Figure 2.

### The nuclear lamina structure and its disintegration in senescence

The nuclear envelope separates nuclear functions from cytoplasmic functions and at its inner surface, provides a docking site for chromatin. The inner nuclear membrane is lined by the nuclear lamina which is composed of a complex meshwork of proteins viz., the type V intermediate filament proteins and the nuclear lamins. Lamins are either A or B type based on sequence homology. In mammals, two major A-type lamins (lamin A and C) and two major B-type lamins (lamin B1 and B2) have been characterized. A-type lamins are derived from one gene (LMNA) by alternative splicing whereas B-type lamins are encoded by different genes [95].

The lamins are composed of a long central α-helical rod domain containing heptad repeats, flanked by globular N-terminal (head) and C-terminal (tail) domains [96]. A CaaX box (where C is cysteine, a is an aliphatic amino acid and X is any amino acid) located at the C terminal of lamins B1 and B2 and lamin A (but not lamin C), undergoes extensive post-translational processing. First, the cysteine residue of the CaaX box is farnesylated and subsequently the aaX is removed by endopeptidases such as RCE1 or ZMPSTE24/FACE1, following which, the cysteine is carboxymethylated. Lamin A undergoes a further maturation step with the removal of 15 amino acids from the C terminus by the enzyme ZMPSTE24 to form mature lamin A [97].

### *Perturbations in nuclear lamina-chromatin interactions*

Loss of lamin B1 is a notable biomarker of senescence in primary human and murine cells [98]. In senescent cells, lamin B1 expression is downregulated at two levels: at the mRNA level [98] as cells exit the cell cycle, and at the protein level, by autophagic degradation [99]. Consequently, lamin B1 is found in conjunction with associated DNA and histones in cytoplasmic chromatin fragments (CCFs). The cytoplasmic chromatin activates the innate immunity arm via cytosolic DNA sensing cGAS-STING and NF-κB pathway leading to both short-term and chronic inflammation by activation of SASP [54,100,101]. A consequence of lamin B1 downregulation is a detachment of chromatin domains normally attached to the nuclear lamina [102] leading to the redistribution of heterochromatin from the nuclear periphery to the interior. This is evidenced by epigenetic profiling of senescent cells which show formation of large-scale domains of H3K4me3- and H3K27me3-enriched mesas and H3K27me3-depleted canyons. Mesas form at LADs in RS and OIS and overlap DNA hypomethylation regions in cancer providing further support that the senescent epigenome precedes cancer progression [58]. Lamin B1 reduction in proliferating cells triggers senescence and the formation of mesas and canyons. The anti-proliferative effect of lamin B1 silencing requires the activation of p53, but not Rb, whereas full induction of premature senescence requires both proteins [103]. Importantly, immunofluorescence studies in liver sections derived from irradiated mice showed lower staining of lamin B1 (but not lamin C) suggesting that it is also a feature of aged cells [98].

### *Laminopathies and link to premature senescence*

Lamins are key proteins linked to premature human aging [104]. The group of heterogeneous diseases caused by lamin dysfunction is called “laminopathies”. Laminopathies among other diseases include HGPS or childhood progeria and Werner’s syndrome or adult progeria, both rare sporadic disorders characterized by accelerated aging [95]. A majority of HGPS patients carry the G608G (GGC>GGT) mutation within exon 11 of lamin A, activating a cryptic splice donor site that results in production of a dominant negative form of a truncated lamin A protein, called progerin that remains farnesylated [105]. With this mutation, HGPS cells exhibit severe abnormalities in nuclear morphology including nuclear blebbing, compromised DNA damage repair, alterations in chromosome organization, abnormal heterochromatin, and accelerated rates of cellular senescence [106]. At the chromatin level, HGPS fibroblasts (made senescent *ex vivo*) show evidence of H3K4me3 mesa formation [58], suggesting a link between abnormal nuclear morphology, premature chromatin changes and accelerated cellular senescence. Additionally, late passage senescent HGPS cells exhibit reduction in H3K9me3 and H3K27me3 levels, and H3K27me3 methylase EZH2 but an increase in another heterochromatic mark, H4K20me3 [62,104]. A recent genome-wide analysis of H3K27me3 in HGPS cells suggested a redistribution of the remaining amount of this modification across the genome that correlates with gene expression changes [107]. Interestingly, progerin is found to accumulate in RS cells [108], and skin cells in the elderly [105] suggesting that it may drive alterations in nuclear morphology, disruption of heterochromatin and downstream gene expression changes in senescence and aging. Werner syndrome patients commonly carry mutations in the WRN helicase. However, a subset of patients may not exhibit WRN mutations but rather carry mutations in the heptad repeats of lamin A (atypical Werner’s). Altered nuclear morphology and mislocalized lamina are also characteristic of these patient-derived aged cells and likely contribute to the accelerated aging phenotype also seen in HGPS [95].

**Figure 3:**
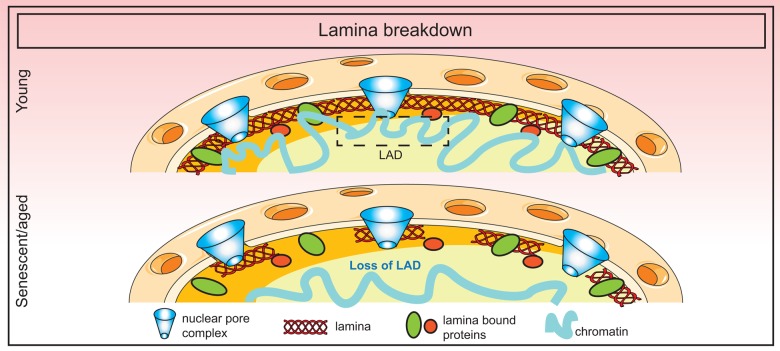
**Breakdown of the nuclear lamina in senescence.** Loss of lamin B1 in senescence triggers the detachment of constitutive heterochromatic regions (lamin-associated domains or LADs) which disorganizes the spatial arrangement of the genome in the nucleus.

The exact cause of lamin B1 downregulation during senescence is unknown although it is speculated that sustained DNA damage may play a role. Alternatively, loss of lamin B1 may be due to the exit from cell cycle coupled to selective autophagy mechanisms as discussed above. Nevertheless, the consequence of this loss is dramatic, affecting lamina structure, nuclear morphology and genome organization. In the case of HGPS patients, some of these detrimental changes can be ameliorated by farnesyltransferase inhibitors that prevent the farnesylation and incorporation of progerin into the nuclear membrane [109,110]. An overall summary of lamina changes in senescence is shown in Figure 3.

### 3D genome (dis)organization in senescence

The genome within the nucleus is hierarchically organized in three dimensions; the first dimension (1D genome) being the linear genome sequence, the second dimension (2D genome) being the arrangement of genes and regulatory elements forming a functional network of the genome, and the third dimension (3D genome) being the spatial organization of the genome in the nuclear space. It has been observed that 3D organization is non-random and that active regions of the genome are segregated from inactive domains. At its highest resolution, the 3D genome is able to define how gene expression is controlled by “looping” of regulatory elements to gene promoters despite being separated in the first dimension [111]. A fourth dimension (4D nucleome), “time”, has been added which integrates the 1D, 2D and 3D information and traces changes in genome organization during processes such as development, adaptation to stress, aging, and disease [112,113].

Higher-order chromatin organization has attracted great attention in recent years due to progress in chromosome conformation capture (3C) methods and high-resolution nuclear microscopy. Using these methodologies researchers elegantly illustrated a top-down view of the 3D genome in which chromosomes are spatially segregated into territories (50-250 Mb), compartments (~5 Mb), topologically associated domains (TADs; ~1 Mb), sub-TADs (0.1-1 Mb) and loops (5-300Kb). Within each territory are clusters of compartments which may be either active (A) or repressive (B). Within each compartment, there are TADs which in turn contain sub-TADs. Sub-TADs contain loops (usually between regulatory enhancers and gene promoters). Each individual structure is physically separated from one another by boundary regions. Three ubiquitously expressed proteins, cohesin, CTCF and YY1, control the organization of loops, sub-TADs and TADs using a loop extrusion mechanism but are dispensable for compartments and territories [114–117]. Condensin II, on the other hand, controls intermixing of territories [118]. Loops and sub-TAD structures are dynamic whereas TADs are largely stable units of transcription and replication but are cell-type specific [119,120].

### *Compartment switching and TAD behavior*

During senescence and aging, there are dramatic changes in the chromatin landscape including changes in histone modification, DNA methylation and nucleosome organization [52], and therefore logically these changes transpire into changes in 3D genome organization [121]. Hi-C experiments in human diploid fibroblast (LF1) cells in deep RS showed (as expected) that compartments and TADs were largely unaltered compared to proliferating or quiescent cells. However, there was a significant decrease in long-range interactions and an increase in short-range interactions. Additionally, a subset of TADs showed compartment switching from B to A (higher frequency) or A to B (lower frequency; cell cycle genes) with gene expression changes in the expected direction. The popular senescence-associated genes such as SASP were in stable A compartments [82]. A higher-resolution Hi-C map in HUVEC, IMR90 and MSC (mesenchymal stromal cells) afforded a finer examination of TAD behavior in senescence whereby ~50% of the TADs were unchanged but the rest showed either a shifting, separating or fusing behavior with the latter being most frequent. HMGB2 was discovered as a novel looping factor found at TAD boundaries that insulates CTCF sites and prevents them from forming long-range interactions. A down-regulation of HMGB2 in senescence prompted the formation of large senescence-induced CTCF clusters (SICCs) [122]. However, this study contradicts the previous study in that the authors found changes in both short- and long-range interactions and limited compartment switching.

In contrast to RS, OIS in WI38 cells showed that long-range cross-boundary interactions were significantly gained but short-range local intra-TAD interactions were lost. This local loss in short-range interactions appeared to occur in regions that correspond to constitutive heterochromatin enriched in H3K9me3, are late replicating, have low GC% and are lamina-associated. The authors thus concluded that they represent regions of heterochromatin disruption. Interestingly, similar changes were also observed in aged fibroblast cells from Progeria patients which are known to undergo nuclear lamina destabilization concomitant with a near complete loss of long-range interactions. In OIS cells uniquely, there was a spatial clustering of the decondensing regions that likely represent SAHFs [123]. These observations while reconciling the observed differences in SAHF formation in OIS (more SAHF) and RS/HGPS (less SAHF) cells, do not fully explain the mechanism of SAHF formation. It is speculated that structures such as SAHFs and SICCs may represent outcomes of liquid phase separation in senescent cells although that remains to be tested.

**Figure 4:**
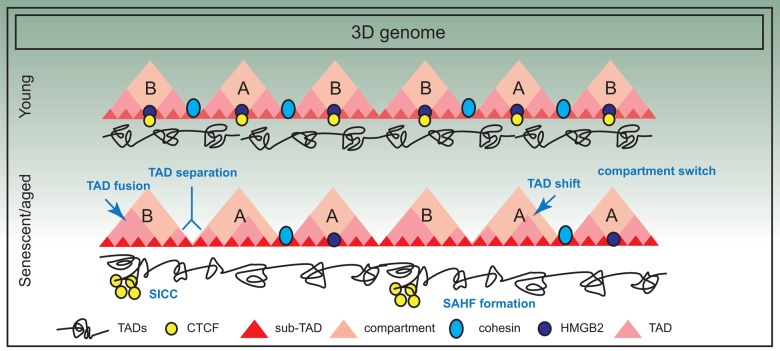
**Three dimensional spatial changes in the genome during senescence.** The 3D arrangement of the genome suffers significant changes in senescence; for example there is evidence of compartment switching, TAD fusing, TAD separation and TAD shifting. Some of these changes are also triggered by decline in chromatin architectural proteins such as HMGB2, a consequence of which is SICC formation. However, in general, TAD structure is maintained.

Orthologous approaches such as chromosome painting, FISH distance measurements, microscopy, FAIRE and DNaseI sensitivity further point to the compaction of chromosomes and increase in overall nuclear volume in senescent cells [124]. Together with a general increase in short-range interactions (in RS and HGPS cells), a collapsing coil model has been proposed [82]. This model envisions that the changes in the 3D genome in senescence and aging manifests in the shrinking of chromosome arms non-homogeneously at different levels of organization. A summary of the frequent alterations in 3D genome organization is presented in Figure 4.

## Future Directions and Translational Perspectives

### The “coming of age” of the senescence field

When cellular senescence was first characterized in *in vitro* cell culture, links to tissue and organismal aging were proposed [1,125]. Critics of cellular senescence questioned its relevance to *in vivo* aging, their possibility of being an artefact and the inevitable lack of senescence despite normal aging in lower organisms. In fact, it was recently discovered that the extremely long lived rodent, naked mole rat, can undergo various forms of cellular senescence that apparently does not affect its longevity [126]. On the contrary, senescence as a pro-aging phenomenon gained popularity with the discovery of biomarkers such as p16 and beta-galactosidase in multiple aged tissues [127]. Mechanistically, the idea of senescent cells being causal in chronic inflammation characteristic of aging, also gained momentum with the discovery of SASP [8].

**Figure 5:**
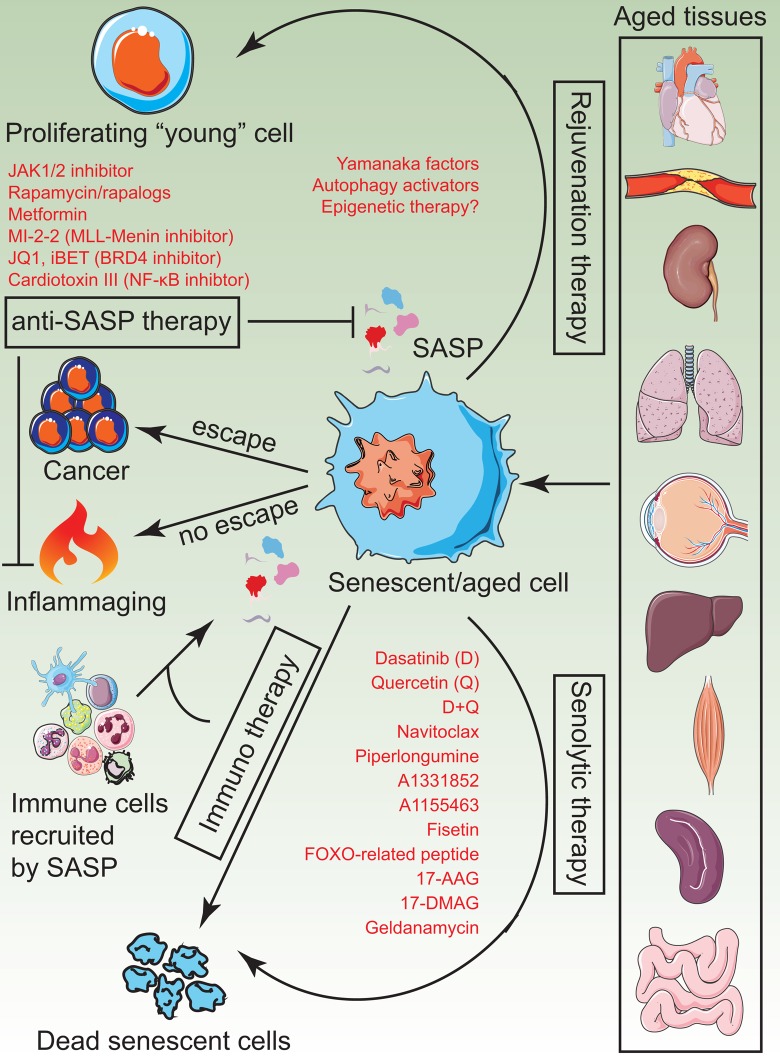
**A visual summary of current senotherapies.** Aged tissues tend to accumulate senescent cells which impose detrimental changes to tissue structure, regenerative ability and physiological function due to chronic inflammation. Current and plausible strategies to treat these adverse effects include administration of senolytics, rejuvenation therapy by induce partial reprogramming to a “youthful” state, anti-SASP therapy to prevent the generation and release of inflammatory cytokines and immunotherapy to activate innate immune mechanisms of the body, which in turn, clear senescent cells naturally.

The senescence field came of age with four major milestones, (a) two proof-of-concept studies showed major improvement in healthspan and lifespan in mice by the targeted ablation of senescent cells [26,27], (b) development of small molecule senolytics as a therapeutic strategy for clearing senescent cells [33], (c) demonstration that senolytics improve physiological function and lifespan in aged mice [34,35,37] and (d) the success of senolytics in pre-clinical studies of a range of age-related conditions [128]. Below, we discuss and illustrate (Figure 5) potential alternatives to senolytics that can deploy epigenetic proteins as “switches” to turn on/off specific pathways in senescent cells for their effective elimination.

### *SASP inhibitors*

Despite the overwhelming success of senolytics, fundamental concerns about specificity and safety prevail. Additionally, the potential benefit of senolytics in treating age-related disease remains to be tested. A second class of molecules that have shown promise in anti-aging rejuvenation therapies is SASP inhibitors. The concept of annihilating the pro-aging arm of senescent cells while preserving the anti-tumor arm is a very attractive treatment option in the elderly who have a high incidence of cancer. Both rapamycin [39] and metformin [129] have shown anti-SASP effects and are on the road to clinical trial for aging. Alternatively, epigenetic enzymes that play a key role in turning on SASP genes (MLL1 [45] and BRD4 [66]) can be inhibited by small molecules to prevent its deleterious effects.

### *Autophagy activation*

Autophagy is a self-degenerative process that clears and recycles damaged cellular components. In a seminal publication, it was reported that basal autophagy is essential to maintain the stem-cell quiescent state while preventing senescence of muscle satellite cells in mice [130]. Furthermore, autophagy declines during aging, calorie restriction activates autophagy, and dysfunctional autophagy is evident in Alzheimer’s disease pathology [131]. Thus, boosting general macroautophagy (non-selective) is a viable anti-aging avenue. The challenge of autophagy-promoting strategies however comes from observations that autophagy of “nuclear” substrates [99,100] might in fact contribute to senescence, aging and inflammation. Selective substrate-specific activation methods (in this case, activation of non-nuclear substrates), need to be developed for use as anti-aging therapy. As an example, selective activation of autophagy directed against damaged mitochondria (mitophagy) that accumulates in senescence and aging has been accomplished [132]. For further development, it is important to understand upstream triggers of macroautophagy and key epigenetic factors that may play in its activation while suppressing nuclear autophagy.

### *Immune-mediated clearance*

Senescent cells are naturally cleared by innate immune mechanisms with the macrophage playing a central role. However, immune cells themselves undergo progressive decline in function (termed immunosenescence) that actively contributes to senescent cell accumulation [14,133]. Furthermore, it has been proposed that subsets of senescent cells become resistant to immune-mediated clearance. Therefore, epigenetic interventions that boost immune surveillance in aged tissues or antibody-based therapies that revert the immune-resistance of senescent cells may also be future rejuvenation strategies [134].

### *Rejuvenation therapy*

Regenerative medicine is a field that provides strategies to repair and restore organ function due to injury, disease and congenital defects. The principles of regenerative medicine can also be applied in aging and age-related disease. Expression of pluripotency factors in senescent cells have been shown to allow cell cycle entry with reset telomere size, gene expression profiles, oxidative stress, and mitochondrial metabolism [42]. Additionally, their expression in mice has also shown amelioration of a panel of age-related phenotypes [43]. Epigenetic factors that can potentiate reprogramming can be used to rejuvenate senescent/aged cells. However, it is important to be cautious with regenerative therapy in the elderly because of its potential to be pro-tumorigenic.

### *Other potential epigenetic therapies*

This review summarizes the major findings of chromatin and nuclear changes in senescent cells (listed in Table 1). The emerging conceptual themes that arise from the observations are (a) a gradual euchromatinization of the genome, (b) loss or disorganization of constitutive heterochromatin due to (c) breakdown of the nuclear lamina and changes in nuclear morphology and (d) loss of spatial organization of the genome. These large-scale changes manifest in profound transcriptional alterations that ultimately activate programs such as SASP and contribute to transcriptional noise. Systematic screens for epigenetic factors will likely yield potential candidates that can be targeted to prevent or reverse the detrimental effects of senescence. Two exemplary discoveries in this field are (a) the discovery of MLL1 (and potentially inhibition of the MLL/Menin interaction) [45] and (b) BRD4 (and potentially BET inhibitors) [66] as direct SASP ameliorating targets. Inhibitors of other epigenetic enzymes, some of which are already in the market for chemotherapy, can be repurposed to provide anti-aging benefits. With the first RNAi therapy being approved by the FDA, the possibilities of epigenetic therapy are limitless.

**Table 1:** Epigenetic themes from studies in senescence in vitro and *in vivo.*

**Theme**	***in vitro* senescence type**	***in vivo* aging**	**Possible therapy**	**Reference**
SAHF with repressive chromatin marks, HP1 and macroH2A	RS, OIS, HGPS			[10,55–57]
SASP	RS, OIS	Multiple aged tissues	Anti-SASP therapy (Fig. 5)	[8,23,44]
Decline in total histone	RS		Boost expression of canonical histones	[53,54]
Canyons and mesas	RS, OIS and HGPS		Histone methylase/demethylase inhibitors	[58]
Increase of H4K20me3	RS, OIS, HGPS	Aged rat liver and kidney		[60–63]
Increase of H4K16ac	RS	Aged human brains	Sirtuin activators	[64,65]
Enhancer formation and score	OIS	Aged mouse heart, liver, cerebellum and olfactory bulb	BRD4 and BET inhibitors	[66,67]
Increase in expression and deposition of histone variants, histone clipping	RS, OIS		Boost expression of canonical histones, Inhibition of cathepsin	[45,57,64,69–71,75]
Global DNA hypomethylation (5mC), focal hypermethylation	RS	Aged mouse liver	TET inhibition	[11,86,87]
SADS	RS, OIS			[82,83]
Derepression of repeat elements	RS	Aged mouse heart, liver, cerebellum and olfactory bulb		[67,84]
Epigenetic clock	RS, OIS (clock predicts cellular age but not senescence)	Multiple human cells and tissues from aged and diseased donors including skin and blood, mouse liver etc.		[87–93]
Loss of lamin B1, nuclear blebs, progerin accumulation	RS, OIS, HGPS	Skin cells from HGPS patients and old humans	Farnesyltransferase inhibitors	[58,98–100,102,103,108–110]
CCF	RS, OIS, DNA-damage induced		Inhibition of unknown endonuclease	[54,99,100]
Compartment switching	RS, OIS, HGPS			[82,122,123]
TAD fusion, separation, shift	RS, OIS, HGPS			[82,122,123]
SICC	RS		Increase expression of HMGB2	[122]

Taken together, anti-aging and senescence-clearing therapies can be devised around many well founded principles and what will likely benefit in the end are combinatorial approaches that rejuvenate senescent cells while preserving its anti-proliferative state and blocking its pro-inflammatory properties. Epigenetic approaches provide tractable solutions in this direction.
